# Optimum fractionation of radiation to combine PD‐1 blockade

**DOI:** 10.1002/mco2.271

**Published:** 2023-05-16

**Authors:** Feifei Teng, Tianwen Yin, Xiao Ju, Peiliang Wang, Yungang Wang, Jinming Yu

**Affiliations:** ^1^ Department of Oncology Tianjin Medical University Tianjin China; ^2^ Cancer Center, Union Hospital, Tongji Medical College Huazhong University of Science and Technology Wuhan Hubei China; ^3^ Department of Radiation Oncology and Shandong Provincial Key Laboratory of Radiation Oncology Shandong Cancer Hospital and Institute Shandong First Medical University and Shandong Academy of Medical Sciences Jinan Shandong China

**Keywords:** anti‐PD‐1, hypofractionation, immunity, radiation

## Abstract

The optimum fractionation of radiation to combine with immune checkpoint blockade is controversial. This study aimed to investigate the fractionated radiation to maximize immunity during combination therapy. To evaluate the abscopal effect, C57BL/6 hPD‐1 knock‐in mice bearing two syngeneic contralateral MC38 murine colon cancer tumors were treated with four distinct regimens of radiotherapy. Three fractions of 8 Gy were chosen as the optimal fractionation to combine with anti‐PD‐1 as the optimal fractionation for maximizing immunity. Anti‐PD‐1 administration enhanced both local and systemic antitumor immunity in a cytotoxic T cell–dependent manner. Meanwhile, the spleen exhibited decreased myeloid‐derived suppressor cells (MDSCs) under combination treatment. Furthermore, RNA‐sequencing revealed significantly increased tumor necrosis factor (TNF) receptors and cytokines associated with lymphocyte infiltration in the combining group. Here we demonstrate that the hypofractionation of 8 Gy × 3f was the optimum‐fractionated dosage to maximize immunity, and the combination of anti‐PD‐1 showed promising results in boosting abscopal effect. Underlying mechanisms may include the activation of T cells and the reduction of MDSCs, which is achieved through the action of TNF and related cytokines. This study indicates a radioimmunotherapy dosage painting method that can be developed to overcome present limitations in tumor immunosuppression.

## Graphical abstract

In this article, the authors discussed the optimum fractionation of radiation maximizing immunity to combine with checkpoint blockade. It indicated that the hypofractionation of 8 Gy × 3f activated antitumor immunity better than conventional fraction and a higher single dose of 20 Gy. Synergistic antitumor effect was shown when combined with PD‐1 blockade in the mouse model. Activated T cells and MDSCs reduction mediated by tumor necrosis factor and associated cytokines are the possible mechanisms. Created with BioRender.com.

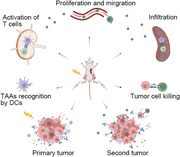

## 1 INTRODUCTION

Radiotherapy (RT) is widely applied for most cancers as a major local treatment modality due to its direct cytotoxic effects on tumor cells. Furthermore, radiation can mobilize antitumor immunity, which is called the abscopal effect,^1^ and plays an important role in the overall efficacy of RT on both targeted and distant metastatic lesions. Many theories have speculated that the abscopal effect may be linked to the fact that RT is able to enhance T cell priming by enhancing the local accessibility of tumor‐associated antigens (TAAs) or by triggering the release of immunostimulatory cytokines.^2^, ^3^ The clinical observations back up these theories; however, the abscopal effect of RT has only occasionally been documented in clinical settings, and the molecular mechanism or pathways underlying it are currently not well understood.^4^, ^5^, ^6^, ^7^ The paucity of evidence that RT can promote therapeutic antitumor immunity systematically makes it restricted to being used as a local tumor treatment in the clinic.

Hypofractionated RT, such as stereotactic radiation therapy (SBRT) and stereotactic radiation surgery, which offer higher dose fractions than conventional fractionated RT and in some tumors ablative, has been routinely employed in various malignant tumors.^8^ Theoretically, larger radiation doses have the potential to cause more rapid cell death, greater vascular damage, and the activation of inflammatory cytokines, which could result in higher peak‐integrated “danger” signals. Meanwhile, abscopal effects have been reported to be greater at moderate fractionated doses in some preclinic studies. However, as hypofractionated RT varies from conventional fractionated RT from a radiobiologic standpoint,^9^ it is yet unknown how the host–tumor immunity is affected by radiation when moving away from the conventional schedule of 2 Gy/fraction, five‐fractions‐a‐week. First, hypofractionated RT delivered higher doses per fraction, as a result of more rapid cell killing and more inflammatory cytokine induction. Second, each treatment session with SBRT typically lasts longer than with conventional fractionated RT because of the interventions made in between beams delivered from various orientations. During this time, various immunogenic tumor cell–killing processes could take place. Third, according to the theory of radiation physics and radiobiologic, the radiobiologic effect of SBRT does not follow the linear‐quadratic model completely. Additionally, the higher delivery of radiation doses brings a great impact on the circulating immune cell system, leading to lymphopenia, potentially undermining the treatment effect.^10^ At this point, only a tiny proportion of patients are currently considered clinically appropriate for hypofractionated RT due to this unfavorable effect.^11^

Combining RT with immune checkpoint blockades recently yielded promising results in some preclinical and few clinical researches.^11^, ^12^, ^13^ As one of the commonly used immune checkpoint blockades, anti‐PD‐1 has a dismally low response rate of less than 30% in patients with advanced‐stage malignancies apart from melanoma.^14^, ^15^, ^16^ In order to successfully produce an antitumor immune response, a variety of specific variables are thought to be required, which may explain the low response rates to anti‐PD‐1. The demands to sensitize T cells to TAAs and the capacity of activated T cells infiltrate to the tumor are the two notable obstacles of anti‐PD‐1 to generate a robust antitumor immune response. When the two therapies are combined, RT can aid in overcoming these obstacles.^14^, ^17^, ^18^ However, this new paradigm is frequently viewed as a medical spectacle without a unified model, and its mechanisms have yet to be elucidated. These considerations were highly relevant to the most advantageous fractionation of RT.

In this study, we chose the C57BL/6 hPD‐1 knock‐in mice that bear MC38 murine colon cancer to examine whether differential fractionated RT to the primary tumor can induce an immune‐mediated abscopal effect in a second tumor beyond the radiation field. We further identified the status of immune cell infiltration in different fractionated RT. Hypofractionated RT of 8 Gy × 3f showed the best effect of primary tumor control with the significantly increased infiltration of CD8^+^ tumor‐infiltrating lymphocytes (TILs) and CD4^+^ TILs, whereas anti‐PD‐1 alone did not have any effect on either tumor control or immune cell infiltration. Then 8 Gy × 3f was chosen to combine with anti‐PD‐1 as the optimal fractionation to maximize immunity as it has the best ability of tumor control at both primary and second tumors. Significantly increased CD3^+^CD4^+^ T cells and CD3^+^CD8^+^ T cells along with decreased MDSCs in the spleen were observed in the combination treatment group, whereas a similar trend was found in lymph nodes with no statistical significance. RNA‐sequencing was used to examine the underlying mechanism of how the combination treatment group effects, a great difference in cytokines and receptors were shown between the combination treatment group and the control group. Overall, our data illustrated that the combination of RT and anti‐PD‐1 augments the treatment efficacy and suggested that the hypofractionation of 8 Gy × 3f may be the optimum fractionation RT regimen to maximize the synergistic antitumor effect. The T cell activation and MDSCs reduction mediated by TNF and associated cytokines may be the possible mechanisms of the combination. This study contributes to the rational design of anti‐PD‐1 and RT combination therapies to maximize responses in cancer patients.

## 2 RESULTS

### 2.1 Fractionated radiation delayed tumor growth

**FIGURE 1:**
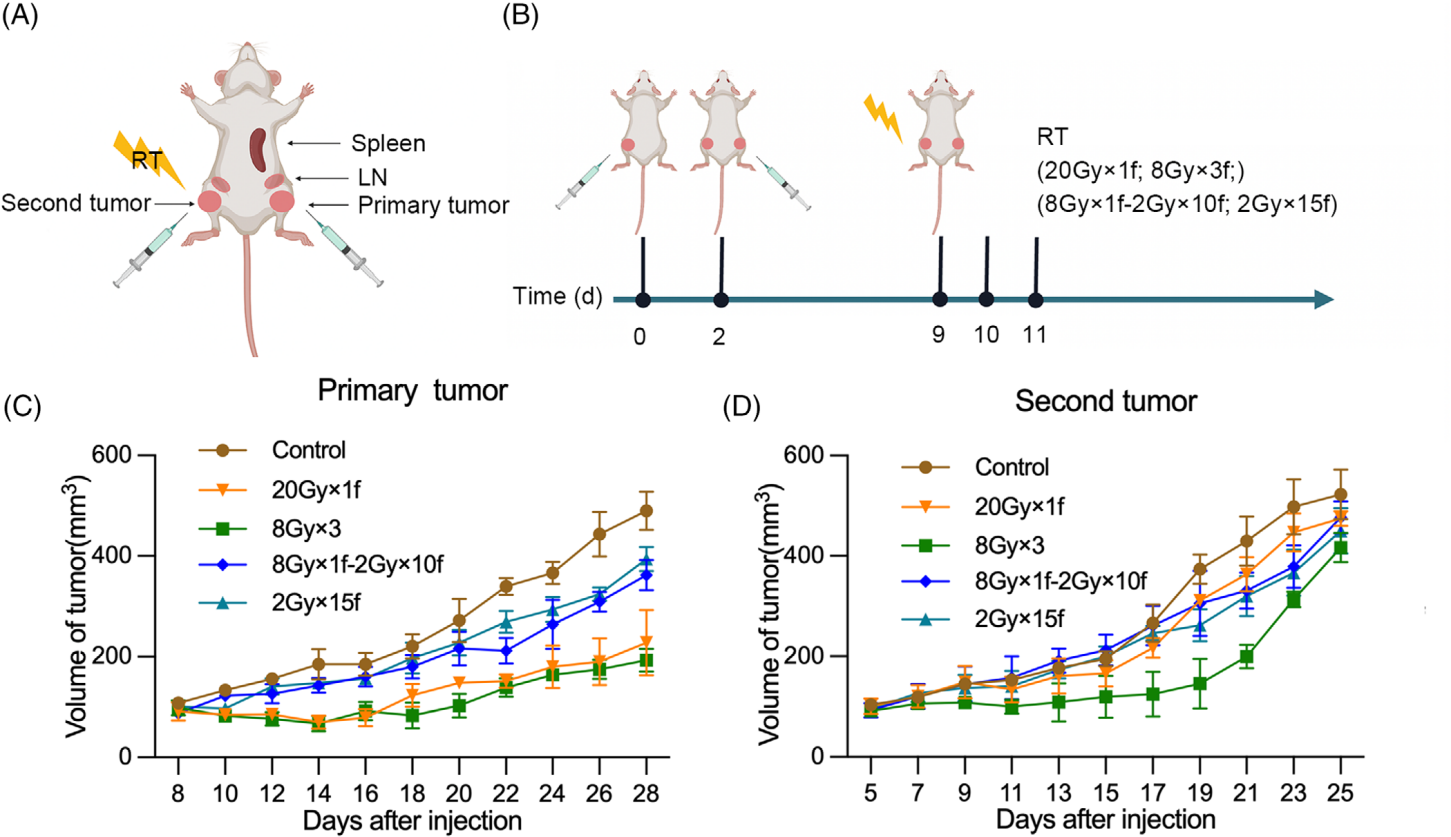
Tumor model and treatment schedule: (A) Mouse models were used to examine the effect of different fractionations of radiotherapy; (B) Mice were implanted with 10^6^ MC38 cells subcutaneously in the left leg (primary tumor) and right leg (second tumor) and treated 9 days later with various fractionations of radiation. (C) Primary tumor size was measured every other day. (D) Second tumor size was measured every other day. *Source*: (A) and (B) Created with BioRender.com.

Different fractionated radiation dosage was designed to find the optimum fractionation of eliciting antitumor effect. Some preclinical evidence has confirmed that hypofractionated RT is more effective than conventional fractionated RT at eliciting abscopal effect. The common hypofractionated pattern “20 Gy × 1f, 8 Gy × 3f” was chosen in accordance with previous study to verify which has the better effect of antitumor immune elicitation between single high dose RT and fractional hypofractionated RT.^14^ However, the negative consequence of increased radiation dose results in the direct destruction of immunological cells, particularly lymphocytes. In this condition, “8 Gy × 1f followed by 2 Gy × 10f” was designed to effectively elicit antitumor immunity along with improved protection for lymphocytes. As the most utilized form of conventional fractionated RT regimen in clinical settings, “2 Gy × 15f” is served as a comparison of different schedules. MC38 grows fast in vivo. By day 28 after injection, the volume of primary control tumors had reached 500 mm^3^, but this was controlled by local radiation (Figure 1A,B). The control effect in primary tumor varied a lot by different radiation fractions. A single dose of 20 Gy and 8 Gy × 3f showed equal effects, which were better than 2 Gy × 15f or 8 Gy × 1f‐2 Gy × 10f followed by eight fractions of 2 Gy on consecutive days (Figure 1C). In the absence of immunotherapy, RT alone did not have any significant effect on the second tumors (Figure 1D). We hypothesized that a single modality of RT mainly elicited local antitumor response in the irradiated primary tumor while is not sufficient for systematic antitumor response.

### 2.2 Three fractions of 8 Gy effectively induce the antitumor immune response

**FIGURE 2:**
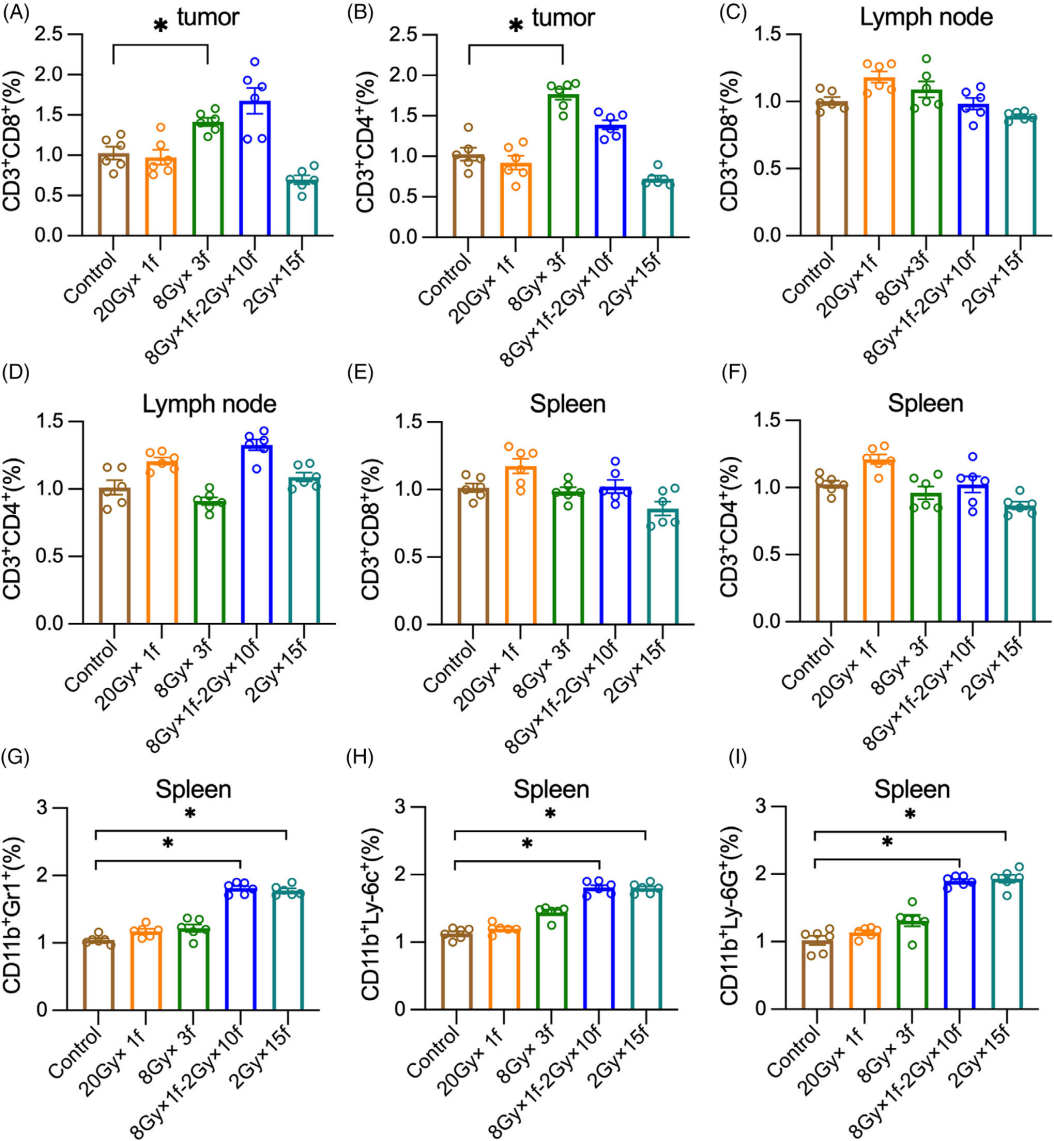
Three fractions of 8 Gy effectively induce the antitumor immune response. Tumor infiltrating lymphocytes (TILs), lymph nodes, and splenocytes were tested 48 h later after radiotherapy. (A and B) Mean CD3^+^CD8^+^ T cells and CD3^+^CD4^+^ T cells as fraction of CD45^+^ T cells in tumors, (C and D) inguinal draining lymph nodes, and (E and F) spleens, (G) CD11b^+^Gr1^+^ myeloid‐derived suppressor cells (MDSCs), (H) CD11b^+^Ly‐6c^+^ MDSCs, and (I) CD11b^+^Ly‐6G^+^ MDSCs as fraction of CD45^+^ splenocytes. *p < 0.05 compared with 0 Gy as control.

It is reported that improved immune cell priming and infiltration are significant processes of how the antitumor treatment effects. Flow cytometry of primary tumor, draining lymph nodes, and spleen were performed to assess the status of immune cell infiltration in these immune organs. Three fractions of 8 Gy increased the infiltration of CD8^+^ TILs and CD4^+^ TILs significantly. A single fraction of 8 Gy followed by 10 fractions of 2 Gy also increased CD8^+^ TILs and CD4^+^ TILs, which were not statistically significant (Figure 2A,B). No significant changes in CD3^+^CD4^+^ T cells and CD3^+^CD8^+^ T cells in the draining lymph nodes and spleen were found in any of the groups (Figure 2C–F). Surprisingly, 8 Gy × 1f‐2 Gy × 10f and 2 Gy × 15f both increased MDSCs (CD11b^+^Gr‐1^+^) in the spleen, which may be related to the weaker effects of tumor control in these two groups by using conventional fractionated RT (Figure 2G–I). Here we demonstrated that the hypofractionation of 8 Gy × 3f was the optimum‐fractionated dosage to maximize immunity.

### 2.3 Fractionated radiotherapy synergizes with PD‐1 blockade in the MC38 colon cancer model

**FIGURE 3:**
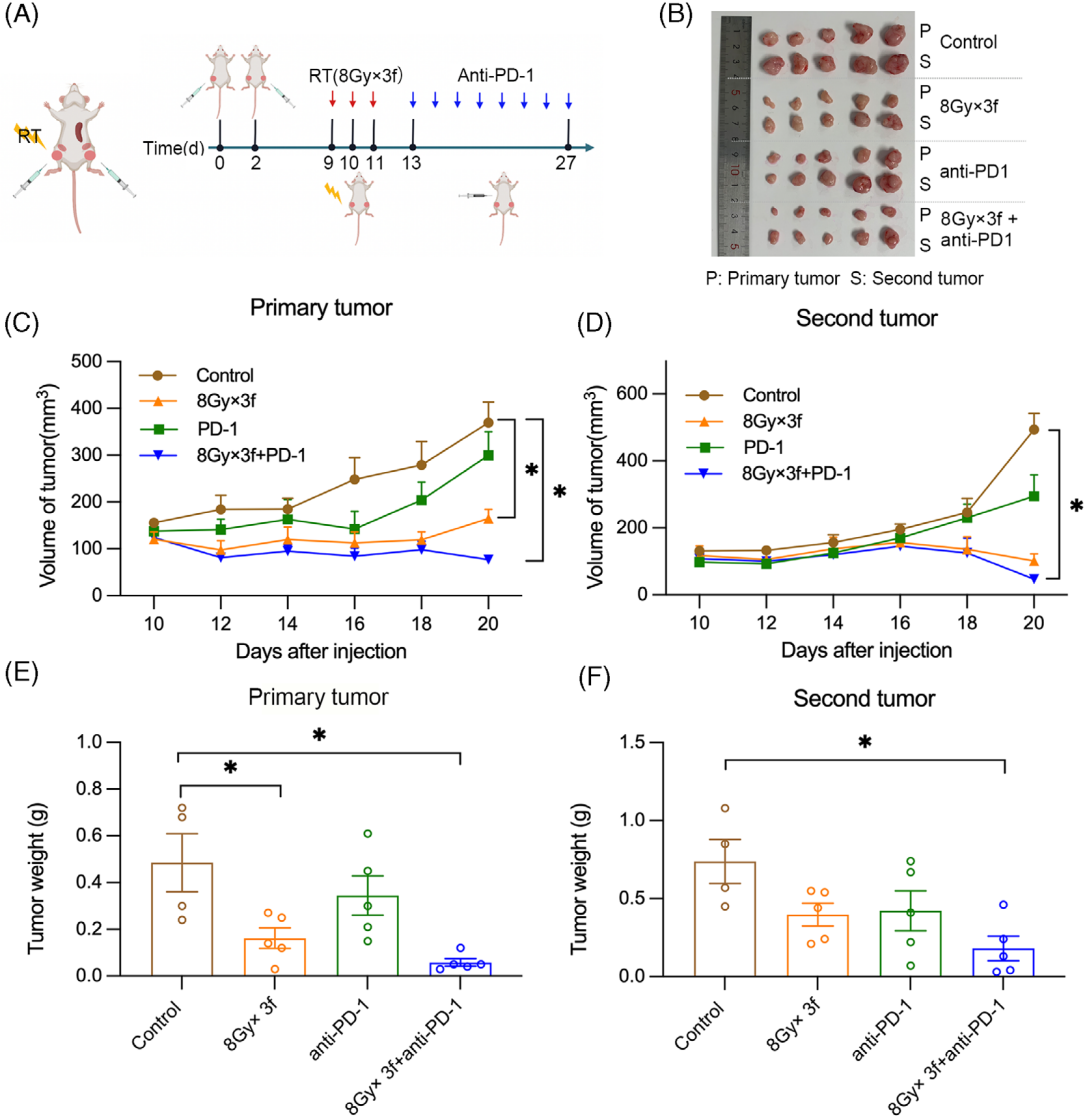
The abscopal effect is induced in MC38 tumor‐bearing mice by the combination of fractionated radiation and anti‐PD‐1: (A) Mouse models were used to test combinations of radiotherapy and immunotherapy. Created with BioRender.com; (B–D) Tumor volume was measured every other day 10 days after injection; (E and F) Mice were sacrificed 20 days after injection, and tumor weight was measured. *p < 0.05 compared with control group.

As three fractions of 8 Gy induced antitumor immunity most effectively, we chose 8 Gy × 3f to combine with anti‐PD‐1 (Figure 3A). As shown in Figure 3, with the absence of RT, anti‐PD1 monotherapy did not show any significant effect either on the primary tumor or on the second tumor. RT as a single modality significantly delayed the primary tumor growth but had no statistically effects on the second tumor. The combination treatment of RT and anti‐PD‐1 effectively controlled tumor growth not only in the primary tumor but also in the second tumor (Figure 3B–F). These data indicated that RT with 8 Gy × 3f induced obvious abscopal effects when combined with PD‐1 blockade.

### 2.4 Fractionated radiotherapy synergizes with PD‐1 blockade‐activated antitumor immune effects

**FIGURE 4:**
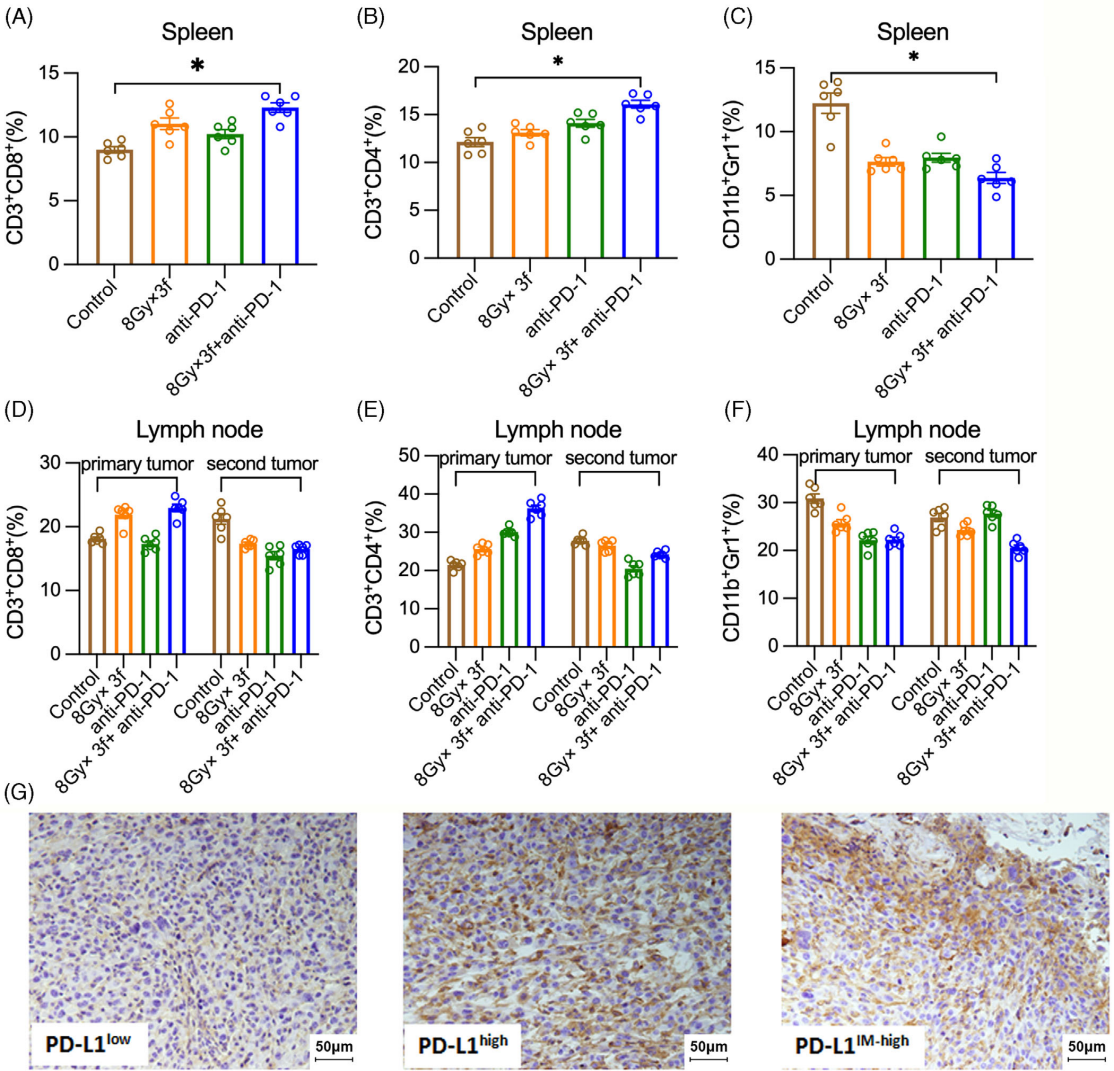
The combination of fractionated radiotherapy with anti‐PD‐1 enhances the T cell infiltration of MC38 tumor‐bearing mice in draining lymph nodes and spleen. Responses in draining lymph nodes and spleens were tested 48 h after treatment: (A–C) Mean CD3^+^CD8^+^ T cells, CD3^+^CD4^+^ T cells, and CD11b^+^Gr1^+^ myeloid‐derived suppressor cells (MDSCs) as fraction of CD45^+^ splenocytes and (D–F) CD45^+^ T cells in draining lymph nodes of the primary and second tumors; (G) Highly intra‐tumor and inter‐tumor heterogeneous PD‐L1 expressions were shown in the tumor tissues. *p < 0.05 compared with control group.

The combination group of 8 Gy × 3f and anti‐PD‐1 exhibited excellent abscopal effects as both the primary and the second tumor almost disappeared after the combination treatment. In this condition, the immune cell infiltration status of draining lymph nodes and spleen were examined by flow cytometry without the assessment of the primary and the second tumors. In the analysis of the spleen, RT combined with anti‐PD‐1 significantly increased CD3^+^CD4^+^ T cells and CD3^+^CD8^+^ T cells with decreasing infiltration of MDSCs, which may be related to the best tumor control and the abscopal effects in the combination treatment group. No significant changes in CD3^+^CD4^+^ T cells, CD3^+^CD8^+^ T cells, or MDSCs were found in the single modality of RT or anti‐PD‐1 group (Figure 4A–C). RT combined with anti‐PD‐1 also showed a trend toward increasing CD3^+^CD8^+^ T cells and CD3^+^CD4^+^ T cells along with decreasing MDSCs in lymph nodes. However, the changes were not statistically significant (Figure 4D–F). It indicated that the antitumor immune effects activated by RT combined with anti‐PD‐1 were mostly performed in the spleen.

We also analyzed the expression and distribution of PD‐L1 in tumor tissue by using immunohistochemistry (IHC) (Figure 4G). Highly intra‐tumor and inter‐tumor heterogeneous PD‐L1 expressions were shown in the tumor tissues. However, we did not find any variation trend in PD‐L1 expressions across all treatment groups.

### 2.5 Gene expression and tumor‐associated signaling pathway changes

**FIGURE 5:**
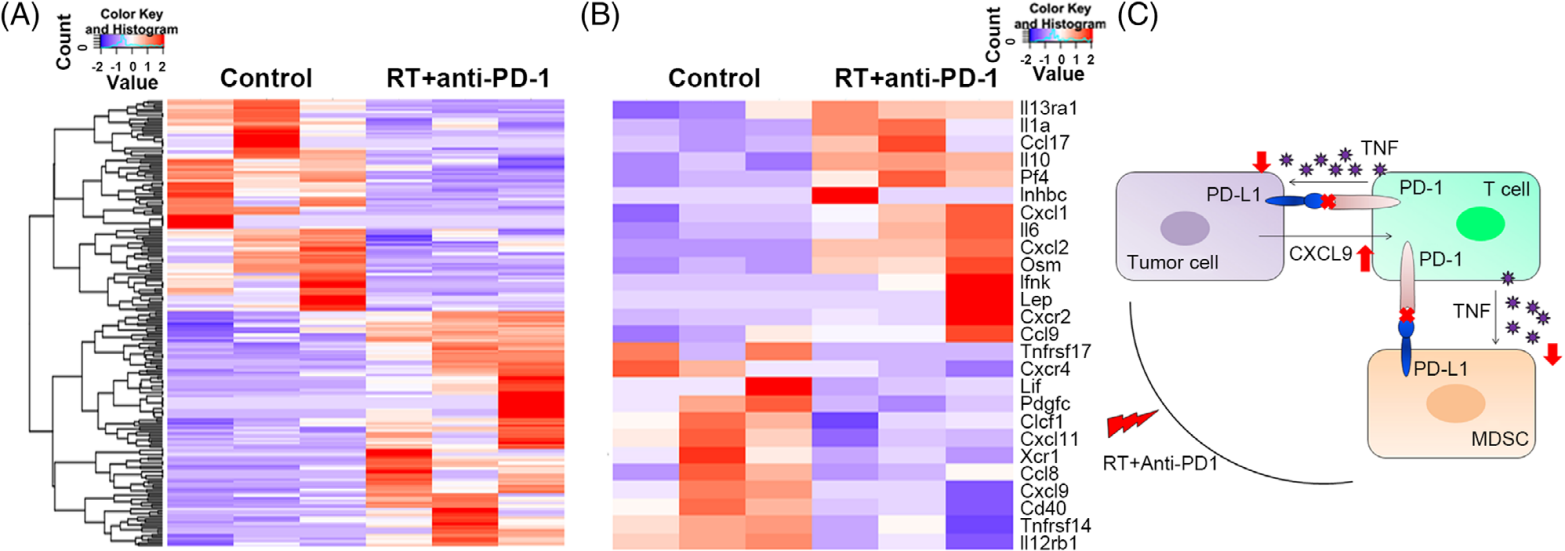
Significant changes of gene expressions are formed by the combination of fractionated radiotherapy (RT) with anti‐PD‐1: (A and B) The significant changes in cytokine and cytokine‐receptor gene expression between the combinatory group and control group; (C) Schematic of potential mechanism for antitumor effect induced by the combination of RT and PD‐1 blockade.

Because the combination of hypofractionated RT and anti‐PD‐1 stimulated antitumor immunity effectively, it was of interest to figure out whether there were changes in antitumor immune‐associated gene expressions. We conducted a tentative exploration of the underlying mechanism of the combination treatment, and the changes in gene expressions were analyzed by RNA sequencing. All significant changes are shown in Figure 5A, where we observed significant changes in cytokine and cytokine‐receptor gene expression that was almost entirely distinct (Figure 5B; *p*‐value <0.05). The tumors that received a combination of RT and anti‐PD‐1 were characterized by lymphocyte infiltration–associated cytokines such as CXCL9 and CXCL11. Additionally, TNF receptors, such as TNFrsf14/TNFrsf17, were activated by the combination treatment. By contrast, tumors in control groups exhibited an increase in general inflammatory cytokines, especially those associated with granulocytic MDSC infiltration, such as CXCL1. This finding indicates that the action of TNF and related inflammatory cytokines, which cause the activation of T cells and reduction of MDSCs, is the underlying mechanism for boosting the abscopal effect of the combination treatment.

## 3 DISCUSSION

In our study, hypofractionated RT as three fractions of 8 Gy was the most beneficial fractionation of RT to maximize antitumor immunity. Moreover, the combination of hypofractionated RT and anti‐PD‐1 immunotherapy brought effective local tumor control and obvious abscopal effects. Activated T cells and reduced MDSCs mediated by TNF and associated cytokines may be the potential mechanisms underlying the combination treatment.

The DNA damage of radiation caused direct tumor cell killing with bare an immune response, the likelihood of effectively handling both local and metastatic disease is dramatically increased when systemic antitumor immunity is triggered in response to radiation‐induced tumor cell death.^19^, ^20^, ^21^, ^22^ In our study, three fractions of 8 Gy, but not conventional fractionation or a single dose of 20 Gy, were immunostimulatory. It is consistent with the findings of previous studies,^19^, ^23^, ^24^ which suggested that hypofractionation may be required for maximizing antitumor immunity. In the meantime, conventional 2 Gy doses led to an increase in MDSCs in spleens. Fu et al.^25^ suggested that the MDSCs decreasing were dependent on CD8^+^ T cells. According to our findings, CD8^+^ T cells had a trend toward decreasing with conventional fractionation, which may account for the emergence of MDSCs and the poor control of tumors by conventional fractionation.^26^ Further studies are needed to clarify it.

Three fractions of 8 Gy increased TILs without increasing T cells in regional draining lymph nodes, indicating that radiation mainly triggered local immunity within tumors. Radiation also induced tumor immunogenic antigen release, which acted as an in situ vaccination, attracting T cells from outside the tumor and actively participating in antitumor immune responses. Previous studies also demonstrated that radiation encouraged the maturation of DCs and the formation of tertiary lymphoid structures (TLS).^27^, ^28^ TLS was found present in the invasive margin and the stroma of tumors, associated with good patient outcomes.^29^, ^30^

C57BL/6 hPD‐1 knock‐in mice were used in our study, which is a suitable model for testing PD‐1 blockade therapy, SHR1210, in a clinical setting. SHR1210 has shown significant benefits in the clinical usage of melanoma, lung cancer, esophageal cancer, and liver cancer. In our study, the combination of RT and anti‐PD‐1 immunotherapy brought effective local tumor control and obvious abscopal effects, which may be mediated by the reduction of MDSCs^31^, ^32^ (Figure 5C). MDSCs have been reported to be correlated with radioresistance and tumor relapse. Our results were consistent with the previous study,^25^, ^26^ which indicated that radiation combined with anti‐PD‐L1 therapy synergistically reduces the local accumulation of MDSCs. Furthermore, the reduction of MDSCs was mediated through the cytotoxic actions of TNF. In this study, we have also observed that TNF receptors were activated by the combination of RT and anti‐PD‐1. However, previous studies have illustrated that TNF plays important roles in promoting the differentiation and survival of MDSCs.^33^, ^34^ The potential explanations for the contradictory effects of TNF on MDSCs may be that TNF is likely to exert differential effects on MDSCs depending on different stages and phenotypes of tumor development.

According to this study, the radiation dose fraction makes a difference in the tumor–host interactions, and this influence extends beyond the irradiated tumor. Radiation can act as an immunological adjuvant with optimal fractionation and shows synergistic effects when combined with immune checkpoint blockade.^35^ However, the study is limited due to a lack of availability of additional cell lines and animal models, as well as the lack of important gene validation, which undermines its reliability. As a tentative exploration of the optimum fractionation of radiation to combine PD‐1 blockade, further preclinical work is needed to confirm the detailed mechanisms underlying the combination treatment. Meanwhile, the rationale of resistance and effective predictive markers are still unclear, a detailed consideration of this new paradigm is required. In conclusion, the combination of hypofractionated RT of 8 Gy × 3f and anti‐PD‐1 treatment exhibited excellent abscopal effect by stimulating CD4^+^ T cells and CD8^+^ T cells, while reducing the accumulation of MDSCs, which may be through the TNF signaling pathway. The different fractionation schedules of RT vary significantly in efficacy and an adequate choice can help enlarge the antitumor effect in the clinic. These findings indicate that the important roles of MDSCs in the tumor immune microenvironment should also be explored in clinical applications under the combination of RT and immunotherapies. This study shed light on the rational design of RT and anti‐PD‐1 combination treatment to enhance responses in cancer patients. Moreover, our findings could provide potential predictive biomarkers for immunotherapies and insight into the designs of new therapeutics.

## 4 MATERIALS AND METHODS

### 4.1 Mice, cell lines, and tumor model

A total of 60 female 6‐ to 8‐week‐old C57BL/6 hPD‐1 knock‐in healthy mice were obtained from the University of Oxford, England and bred in Shanghai Laboratory Animal Center, CAS (SLACCAS), Shanghai. Mice were maintained under specific pathogen‐free conditions. All mice were assigned to various treatment groups as indicated randomly, and every group contained six mice. The mouse model contains a chimeric sequence of PD1, in which exon 2 of the mouse Pdcd1 gene is replaced with the human counterpart. This study was approved by the Ethics Committee of Shandong Cancer Hospital. MC38 is a colon adenocarcinoma cell line, provided by Jiangsu Hengrui Medicine company and cultured in RPMI‐1640, supplemented with 10% FCS and 1% penicillin‐streptomycin (Gibco) at 37°C and 5% CO_2_. Mice were injected subcutaneously with 1 × 10^6^ MC38 cells, respectively, in the left thigh on day 0 (primary tumor) and in the right thigh on day 2 (second tumor). Tumor growth was calculated by measurements in two vertical dimensions every other day. The tumor volume (mm^3^) was calculated from the following formula: volume = (length × width^2^)/2. The Animal Care and Use Committee of Shandong Cancer Hospital authorized the animal experiments.

### 4.2 Radiation and immunization treatment

Radiation was applied when the tumor grew approximately 8 mm in diameter. Mice were anesthetized and positioned on a platform with lead shielding the body, except for the left leg, which was irradiated with a dose rate of 1.84 Gy/min at 300 kV and 10 mA using a Gulmay RS320 X‐ray unit filtered (Gulmay Medical LtD., Camberley, Surrey, UK). The X‐rays were administered vertically focused on the surface at a distance of 20 cm. Mice received a single dose of 20 Gy (20 Gy × 1f), 3 fractions of 8 Gy (8 Gy × 3f), 15 fractions of 2 Gy (2 Gy × 15f), or a single fraction of 8 Gy followed by 10 fractions of 2 Gy in consecutive days (8 Gy × 1f‐2 Gy × 10f). PD‐1 blocking mAb SHR1210 (Hengrui Medicine company, Jiangsu, China) or vehicle (PBS) was administered by intraperitoneal injection at a dose of 10 mg/kg (200 μg/mouse) every day for a total eight times after the second day of RT finished. Tumor growth was evaluated every other day. Two days (48 h) after treatment finished, mice were euthanized by exsanguinations. The tumors, regional draining lymph nodes, and spleens were isolated and weighed.

### 4.3 Flow cytometry

Tumor tissues were digested by 1 mg/mL collagenase type IV (Sigma, USA) and 0.2 mg/mL DNase type I (Sigma, USA) for 30 min at 37°C. Single‐cell suspensions of the spleen, lymph nodes, and tumor were obtained. Cells were blocked with anti‐FcR (Biolegend, USA) and then stained with antibodies against CD3, CD8, CD4, CD11b, Ly6G, and Ly6C (Biolegend, USA). All the samples were collected on a FACS Calibur Flow Cytometer (BD, USA). The data were analyzed by using FlowJo software (Tree Star Inc., USA).

### 4.4 Immunohistochemistry

Tumors were harvested and fixed in 4% paraformaldehyde for 1 h, followed by incubation in 30% sucrose overnight and then frozen at optimal cutting temperature. IHC was performed by using standard automated protocols.^36^ After deparaffinized with xylene and graded alcohol, antigen retrieval was performed by microwaving under high pressure. Nonspecific binding was blocked with goat serum for 1 h and then incubated with PD‐L1 primary antibodies (10 μg/mL, Abcam, USA.). Subsequently, slides were incubated with a secondary antibody. Finally, sections were counterstained using hematoxylin, followed by dehydration, and mounted with a cover slip.

### 4.5 RNA‐sequencing

Total RNA was extracted from the tumor samples, and the quality was checked on an Agilent Bioanalyzer 2100 RNA 6000 Nano Kit (Agilent Technologies, USA). TruSeq RNA Sample Prep Kit v2 and the Hiseq2000 Sequencing System (Illumina, USA) were used for library generation and sequencing according to Illumina protocols.

By using the TopHat2 short read alignment program, All the reads were realigned to the mouse genome assembly in a version of the UCSC mm10. Subsequently, the adaptor sequences were removed, and the low‐quality sequences were trimmed with cut adapt. By using TopHat2 with parameter −*r* 50, the reads were mapped to the genome. The mRNAs for all annotated genes were calculated by using the software package HTseq (version 0.6.1) (http://sourceforge.net/projects/htseq/website). The normalization and the model fitting were analyzed in R. The edgeR Bioconductor package was used for upper quartile normalization and the negative binomial exact test. The genes were considered to be differentially expressed with *p* values and FDR values <0.05, and fold change ≥±1.5.

### 4.6 Statistical analysis

Data were analyzed using Statistical Product and Service Solutions (IBM SPSS17.0). Data were represented as the mean ± SEM for all figures in which error bars were shown. The *p* values were assessed by using unpaired Student *t*‐tests. The *p*‐values of less than 0.05 were considered statistically significant.

## AUTHOR CONTRIBUTIONS

Feifei Teng and Jinming Yu designed the study. Xiao Ju and Peiliang Wang provided the data and materials. Tianwen Yin and Yungang Wang drafted the article. All authors read and approved the final manuscript.

## CONFLICT OF INTEREST STATEMENT

All authors have read the journal's authorship agreement and policy on the disclosure of potential conflict of interests. There was no conflict of interest.

## ETHICS STATEMENT

The study was approved by the Ethics Committee of Shandong Cancer Hospital (reference number: SDTHEC2023003110).

## Data Availability

The datasets during and/or analyzed during the current study are available from the corresponding author upon reasonable request.
